# A Novel, Safe, Non-Adjuvanted Alphavirus Replicon-Based Vaccine Expressing the Feline Leukemia Virus Envelope Protein Protects Against Virulent FeLV Challenge

**DOI:** 10.3390/vaccines13070697

**Published:** 2025-06-27

**Authors:** Kari Carritt, Randall Davis, Ken Stachura, Paige Crumley, Mark Mogler, Madeleine Stahl, Lijuan Deng, Zach Xu, Ian Tarpey

**Affiliations:** 1 Research and Development Department, Merck Animal Health, Elkhorn, NE 68022, USA; 2 Research and Development Department, Merck Animal Health, Ames, IA 50010, USA; 3 Veterinary Insights and Medical Affairs, Merck Animal Health, Rahway, NJ 07065, USA; 4 Global Statistics and Data Management, Merck Animal Health, Elkhorn, NE 68022, USA; lijuan.deng@merck.com; 5 Research and Development Department, MSD Animal Health, 5831 AN Boxmeer, The Netherlands

**Keywords:** feline leukemia virus, RNA particle, envelope protein, vaccine, efficacy, safety

## Abstract

**Background/Objectives:** A number of different vaccines against feline leukemia virus (FeLV) are available; however, there is continuous debate regarding the efficacy advantages of adjuvanted vaccines versus the potential safety advantages of non-adjuvanted vaccines. **Methods:** For this reason, we developed a non-adjuvanted vaccine based on a replicon RNA particle (RP) expressing the FeLV gp85 envelope protein, which possesses the safety of a non-adjuvanted vaccine while consistently providing high efficacy. **Results:** In two efficacy studies, a high-level of protection against virulent FeLV challenge was demonstrated with two doses given 3 weeks apart based on the prevention of FeLV p27 antigenemia. Furthermore, in both studies, we compared this novel vaccine against a non-adjuvanted, canarypox-vectored FeLV vaccine, demonstrating that none of the cats that received two doses of the RP-FeLV vaccine developed persistent antigenemia post-challenge. In comparison, of cats receiving the canarypox-vectored FeLV vaccine, three of seven (43%) became persistently antigenemic in one study, and three of ten (30%) became persistently antigenemic in the other study. In a field safety study using two commercial serials, safety of the RP-FeLV vaccine was demonstrated in over 800 cats receiving two doses of the vaccine. **Conclusions:** These data suggest that the RP-FeLV vaccine offers advantages over some current FeLV vaccines by combining the safety profile of a non-adjuvanted vaccine with the induction of a robust immune response demonstrated by some adjuvanted vaccines.

## 1. Introduction

Feline leukemia virus is a gamma retrovirus transmitted readily via the oronasal route. Infection of cats can manifest itself in a wide variety of clinical disorders, including immunosuppression, inflammatory disease, and lymphoid or myeloid tumors [1]. The extent of the disease is dependent on a multitude of factors including age, FeLV strain, viral load upon exposure, route of infection, plus host immune status. Despite the availability of point-of-care tests and vaccines, FeLV prevalence as detected by p27 antigenemia is still common, especially in non-vaccinated populations [2,3,4,5]. FeLV infection can be categorized as an abortive, progressive, regressive, or focal disease [6,7] related to the ability of the immune response to control the infection. Abortive infection is characterized diagnostically by negative tests for p27 antigen (ELISA), RNA, and proviral DNA (PCR). Abortive cats are able to clear the infection and prevent viremia. Progressive infection is characterized by positive tests for the p27 antigen, RNA, and proviral DNA. In cats with progressive infection, FeLV replicates and circulates for a prolonged period, the cats are capable of spreading FeLV to other cats, and a terminal outcome due to FeLV infection is typical. Regressive disease is characterized diagnostically by negative p27 antigen tests and RNA but positive tests for proviral DNA. Focal infections are considered rare and may result in discordant test results [6,8]. Originally characterized by detection of viremia based upon virus re-isolation, immunofluorescence, and/or p27 protein antigenemia, FeLV diagnosis is now recommended to include PCR to detect non-viremic, provirus-positive cats [9].

FeLV-induced disease can largely be prevented by vaccination. There are a number of FeLV vaccines currently available based on a variety of different FeLV antigens, different vaccine technologies, and the presence or absence of adjuvants. Most FeLV vaccines contain inactivated, whole FeLV virus particles or subunit FeLV proteins formulated with adjuvants such as aluminum hydroxide, saponins, or block polymers [10,11,12,13]. The function of the adjuvant is to enhance the immunogenicity of the antigen, usually by inducing an inflammatory response, which activates the innate immune response, attracting key antigen-presenting cells, which in turn stimulate the adaptive immune response [14]. FeLV vaccines also can contain excipients such as stabilizers, culture material, and preservatives; therefore, the vaccine composition can vary considerably between manufacturers. While most of these adjuvanted vaccines perform well in terms of efficacy, only one adjuvanted vaccine has demonstrated a duration of immunity of two years after the initial vaccination course [15]. However, there continues to be debate with regard to the potential induction of injection site fibrosarcomas with adjuvanted vaccines [16,17,18]. Though the number of fibrosarcomas diagnosed are relatively low, a non-adjuvanted vaccine was developed based on a live, recombinant canarypox virus vector (Purevax^®^ Recombinant FeLV) [19]. This vaccine has been well received in terms of safety, and it is used broadly; however, concerns have been raised regarding the efficacy of the canarypox-vectored FeLV vaccine. While some studies have demonstrated comparable efficacy to adjuvanted feline leukemia vaccines [20,21], other studies have demonstrated sub-optimal performance compared to certain adjuvanted vaccines [10,12]. Furthermore, there remains continuing debate regarding the experimental design of the comparative FeLV vaccine trials, including vaccination regimes, challenge strain, route of challenge, and the use of immunosuppression to ensure uptake of virulent FeLV virus.

In the development of this novel FeLV vaccine, we utilized an Alphavirus-based replicon technology derived from the attenuated TC-83 strain of Venezuelan equine encephalitis virus (VEEV) to express the FeLV envelope protein gp85. Replicon technology has been tested in numerous species [22,23] and has been shown to be safe and efficacious in cats with a variety of antigens, including the SARS-CoV-2 spike protein [24]. The replicon forms the basis of the Sequivity^®^ RNA Particle vaccine platform, which is currently licensed in the US for multiple swine applications. In this system, the foreign gene of interest, in this case FeLV envelope protein gp85, is inserted in place of VEEV structural genes, generating a self-amplifying RNA capable of expressing the gene of interest upon introduction into cells. Removing the structural VEEV genes renders the RNA particle propagation-defective. Upon vaccination, the RNA particles deliver the replicon RNA to the cells. The structural glycoproteins of the RNA particle target and bind to dendritic cells, which have been shown to stimulate a balanced and robust adaptive immune response [25]. Importantly and in contrast to viral vectors based on abortive replication cycles, the RNA is self-amplifying, directing the translation of up to 15–20% of the total protein expression in cells [26] and providing the opportunity to induce long lasting immunity. Importantly, RNA particle-based vaccines have been shown to induce both innate and adaptive immune responses, including virus neutralizing antibodies and T cell responses [27], circumventing the requirement for an adjuvant. Furthermore, they do not spread, cannot revert to virulence, and can be used to differentiate infected from vaccinated animals (DIVA) where required. In addition, the RNA particle vaccine platform allows for vaccine production with reduced serum and without the use of antibiotics, preservatives, or thimerosal. The RNA particle FeLV vaccine is marketed by Merck Animal Health, Rahway, NJ, USA, under the tradename Nobivac^®^ NXT FeLV. A dose-ranging study was conducted in order to determine the minimum protective dose as well as comparison studies and a field safety study.

## 2. Materials and Methods

### 2.1. Vaccine Formulations

The gp85 envelope gene of the Glasgow-1 FeLV subtype A virus (GenBank accession No. P08359) was used for the RP-FeLV envelope construct. The open reading frame of FeLV envelope glycoprotein was codon-optimized and synthesized with flanking AscI and PacI restriction sites (ATUM, Newark, CA, USA). A TC-83-derived replicon vector containing the codon-optimized gene was constructed as described previously [24]. Production of TC-83 replicon RNA particles was conducted similarly to methods previously described [22,24]. The RNA particles were eluted in a 400 mM NaCl + 5% sucrose (*w*/*v*) buffer, passed through a 0.22 micron filter, and aliquoted prior to downstream formulation and testing. As a control, RNA particles expressing green fluorescent protein (GFP) were also prepared as described above.

The RP-FeLV vaccines for the dose-ranging study were in a liquid formulation of 1 mL, as this was a preliminary study to assess the dose response of the RNA particle prior to formulation studies. The RP-FeLV vaccines for the comparison and field safety studies were commercial serials lyophilized, stored at 2–8 °C, and reconstituted in 0.5 mL of vaccine diluent prior to use. The titers of functional RP-FeLV or RP-GFP vaccine were determined by immunofluorescence assay on infected Vero cell (Alphavax Inc., Durham, NC, USA) monolayers. Briefly, the vaccines were serially diluted and added to a Vero cell monolayer culture in multi-well plates and incubated at 37 °C for 18–24 h. After incubation, the cells were fixed and stained with the primary antibody (anti-gp85 virus glycoprotein or anti-VEEV non-structural protein (nsp) 2) followed by a fluorophore-conjugated species-specific secondary antibody. RNA particles were quantified by counting all positive, fluorescent stained cells in 2 wells per dilution.

For the dose-ranging and comparison studies, a commercial serial of non-adjuvanted, canarypox-vectored FeLV vaccine (Purevax^®^ Recombinant FeLV, Boehringer Ingelheim Animal Health Inc., Athens, GA, USA) was included. A different serial was used for each of the two studies: the serial used for the dose-ranging study was a 1 mL dose presentation, and the serial for the comparison study was a 0.5 mL dose presentation. The serials were within expiration dating and used as per the manufacturer’s instructions.

### 2.2. Challenge Virus

For the dose-ranging and comparison study, FeLV 61E (subtype A) prepared by passage in cats was administered on four occasions, every other day. Cats were lightly sedated, and 5.8 Log_10_ PFU of challenge virus was administered via the oronasal route (dose-ranging study) or 3.0 Log_10_ FAID_50_ via the intraperitoneal route (comparison study). The amount of virus inoculated was back-titrated on Crandell-Rees Feline Kidney (CRFK) cells by either plaque assay or endpoint dilution assay each day after use.

### 2.3. Sample Collection

For the dose-ranging study, blood samples were collected weekly in serum separation tubes (SSTs) between three- and twelve-weeks post-challenge, and serum was separated from red blood cells and stored frozen for testing by p27 antigen ELISA. For the comparison study, blood samples were collected weekly in EDTA tubes between three- and twelve-weeks post-challenge, and plasma was separated by centrifugation and stored at 2–8 °C for p27 antigen ELISA and RT-PCR testing. Whole blood was stored at 2–8 °C for PCR testing.

### 2.4. FeLV p27 ELISA

FeLV p27 antigen testing on serum and plasma samples was performed by ELISA (ViraCHEK FeLV^®^, Zoetis, Parsippany, NJ, USA) following the manufacturer’s instructions. Briefly, 50 µL of sample was added to anti-p27 antigen-coated wells; then, 50 µL of horseradish peroxidase conjugate was added to each well. Plates were incubated for 5 min at 15–30 °C, then washed five times with a wash buffer. TMB substrate (100 µL/well) was added, and the plate was incubated for five minutes at 15–30 °C. Following color development, 50 µL/well of stop solution was added. The absorbance of each well was measured at 650 nm; samples were considered positive if the ratio of sample OD/positive control OD (s/p ratio) was greater than or equal to 0.100.

### 2.5. PCR

FeLV proviral DNA were quantified using real-time PCR (qPCR) (Zoologix, Westlake Village, CA, USA). The PCR was performed on DNA extracted from whole blood samples using the QIAGEN^®^ DNeasy^®^ Blood & Tissue Kit (QIAGEN, Germantown, MD, USA). The assay detects but does not differentiate FeLV types A, B, and C. Endogenous FeLV sequences are not detected. The limit of detection is approximately 400 copies of target sequence per 1 mL of blood. The PCR primers and methods are as previously described [28].

### 2.6. RT-PCR

FeLV plasma viral RNA was quantified using real-time reverse transcription PCR (RT-qPCR) (Zoologix, Westlake Village, CA, USA). The RT-qPCR was performed on RNA extracted from the plasma fraction of blood samples with the QIAGEN^®^ QIAamp^®^ Viral RNA Mini Kit (QIAGEN, Germantown, MD, USA). The assay detects but does not differentiate FeLV types A, B, and C, and endogenous FeLV sequences are not detected. The limit of detection is approximately 1000 copies per 1 mL of plasma. The PCR primers and methods are as previously described [28].

### 2.7. Total Protein Assay

Total protein of commercial vaccines was quantified with the Pierce™ BCA protein assay kit (Thermo Fisher Scientific, Waltham, MA, USA). The assay uses bicinchoninic acid (BCA) for colorimetric detection of the reduction of copper cation by protein. Samples and standards were incubated with the BCA reagent at 37 °C for 30 min, and the optical densities of standards and test samples were measured at a wavelength of 562 nm. The optical density of the test samples was compared to a standard curve of bovine serum albumin (BSA).

### 2.8. Bovine Serum Albumin Assay

The bovine serum albumin (BSA) content of commercial vaccines was quantified with a BSA enzyme-linked immunosorbent assay (ELISA) kit (Cygnus Technologies, Leland, NC, USA). The assay uses two antibodies specific for BSA: a capture antibody coated on microplate strips and the detection antibody labelled with horseradish peroxidase (HRP). Samples and standards were tested in duplicate. Standards and test samples (undilute and diluted 1:4) were incubated in the microplate at 23 °C for one hour on an orbital plate shaker. Following washing of test strips, TMB dye substrate was added to each well, and the microplate was incubated static for thirty minutes. After the addition of the stop solution, the plate was read on a microplate spectrophotometer (SpectraMax^®^ 190, Molecular Devices, San Jose, CA, USA) at a test wavelength of 450 nm and reference wavelength of 650 nm. Optical density of test samples was compared to a standard curve of BSA using a linear curve fit. The optical density is directly proportional to the concentration of BSA.

### 2.9. Data Analysis

Persistent FeLV antigenemia was defined as the presence of the FeLV p27 antigen in serum, detected by ELISA, for three consecutive weeks or on at least five occasions, consecutive or not, between the 3rd and 12th week post-challenge. This definition follows the United States Department of Agriculture (USDA) criteria for licensing studies for FeLV vaccines [29].

The preventable fraction (PF), which is used to express vaccine efficacy, was calculated as (percent of persistent antigenemia in controls—percent of persistent antigenemia in vaccinates)/percent of persistent antigenemia in controls × 100% [13].

Statistical analysis was performed using R Statistical Software version 3.5.3 and SAS (Statistical Analysis System) version 9.4. Preventable fraction and associated 95% confidence intervals were calculated using the RRtosst function in R-3.5.3. The proportion of cats positive for DNA and RNA in each group was calculated and compared using Fisher’s Exact Test. Statistical significance was declared at a two-sided *p*-value < 0.05.

### 2.10. Dose-Ranging Study Design

Purpose-bred cats, 8–9 weeks of age, were blocked by litter and randomly assigned to treatment groups of 8 cats each. All cats tested negative for FeLV infection by p27 ELISA prior to vaccination and challenge. All personnel testing laboratory samples were blinded to the treatment groups. Cats were vaccinated subcutaneously with a dose of either 2.5 × 10^8^ RP/dose (Group 1) or 3.6 × 10^7^ RP/dose (Group 2) RNA particle FeLV vaccine. One group of cats was vaccinated with a commercial serial of canarypox-vectored FeLV vaccine according to the manufacturer’s directions (Group 4). An additional group of cats was vaccinated with a single dose of 1.5 × 10^8^ RP/dose RNA particle FeLV vaccine (Group 3) at 11–12 weeks of age. A group of 8 cats served as controls and were vaccinated with a placebo vaccine consisting of Minimum Essential Medium (MEM) (Group 5) (Table 1). During the vaccination phase, Groups 1, 2, and 3 were housed together in one room and Groups 4 and 5 were each housed in individual rooms. During the challenge phase, all cats were comingled in the same room. Prior to challenge, one cat in Group 4 was removed from the study for health reasons not associated with the vaccine. All remaining cats were challenged with virulent FeLV via the oronasal route four times over eight days, starting four weeks after the second (or single) dose of vaccine, and bled weekly between three- and twelve-weeks post-challenge to monitor p27 antigenemia.

### 2.11. Comparison Study Design

Purpose-bred cats, 8 weeks of age, were blocked by litter and randomly assigned to 3 treatment groups of 10 cats each. All cats tested negative for FeLV infection by p27 ELISA prior to vaccination and challenge. All personnel testing laboratory samples were blinded to the treatment groups. Cats were vaccinated subcutaneously as per the manufacturer’s instructions with a commercial serial of either RNA particle FeLV vaccine (Group A) or canarypox-vectored FeLV vaccine (Group B). A third group of cats were vaccinated with a placebo vaccine (sterile 0.9% saline solution) (Group C). During the vaccination phase, cats in Groups A and C were housed together in one room and cats in Group B were housed in another room to prevent potential shedding of the live vaccine. For the challenge phase, litters were randomly assigned to one of two rooms, and cats from all 3 treatment groups were commingled in the two rooms. Starting three weeks after the second dose of vaccine, all cats were challenged with virulent FeLV via the intraperitoneal route four times over seven days. All cats were bled weekly between two- and twelve-weeks post-challenge to monitor p27 antigenemia, proviral DNA by PCR, and viral RNA by RT-PCR.

Samples of the two commercial vaccine serials were each rehydrated in 0.5 mL of the accompanying diluent, as per the label instructions, and tested for total protein and bovine serum albumin content.

### 2.12. Field Safety Study Design

A total of 837 cats were vaccinated with one of two commercial serials of RNA particle FeLV vaccine, formulated at a typical field dose in 0.5 mL. The cats were a mixture of client-owned and purpose-bred cats from 7 different sites in 6 states representing a variety of breeds. A total of 378 cats were 8 weeks of age, 63 cats ranged from 9 weeks to 11 months of age, and 396 cats ranged in age from 1 year to 16 years. Of the study animals enrolled, 820 cats were administered two doses subcutaneously approximately 3 weeks apart, and 17 cats were administered one dose subcutaneously. The cats were observed daily for any adverse reactions from the time of the first vaccination through 14 days post-booster vaccination.

## 3. Results

### 3.1. Dose-Ranging Study

Efficacy of the RP-FeLV vaccine administered at different doses and/or with different dosing schedules was evaluated compared to a commercial serial of canarypox-vectored FeLV vaccine based on persistent antigenemia. The placebo control group exhibited 88% (7/8) persistent antigenemia, indicating a robust challenge in the absence of immunosuppression (Table 2). At both dose levels of RP-FeLV vaccine, no cats became antigenemic at any point during the study, with 100% (8/8) of cats protected in both RP-FeLV groups (Groups 1 and 2). In the group receiving a single dose of RP-FeLV, only one cat exhibited persistent antigenemia for a preventable fraction of 86%. In contrast, 43% (3/7) of cats became persistently antigenemic in the group vaccinated twice with the canarypox-vectored FeLV vaccine, resulting in a 51% preventable fraction.

### 3.2. Comparison Study

Commercial serials of RP-FeLV and canarypox-vectored FeLV vaccines were compared in a vaccination challenge study. Based on FeLV p27 ELISA results, all of the control cats became persistently antigenemic, indicating a robust challenge without immunosuppression. All of the cats (10/10) in the RP-FeLV group were protected from persistent antigenemia, while all of the cats (10/10) in the placebo group were affected with persistent antigenemia. The preventable fraction of the RP-FeLV vaccine was 100%, i.e., all of the disease in the placebo group could have been prevented if cats received the RP-FeLV vaccine (95% confidence interval [69%, 100%]). A total of 70% (7/10) of cats in the canarypox-vectored FeLV group were protected from persistent antigenemia (preventable fraction 70%, with 95% confidence interval [35%, 93%]) (Table 3). One cat in Group B (canarypox-vectored FeLV) was removed during the challenge phase due to enlarged lymph nodes caused by FeLV infection; this animal had already been confirmed as persistently antigenemic.

All of the cats in the placebo group had detectable levels of proviral DNA at all testing timepoints (weeks 2 through 12 post-challenge). A lower incidence of proviral-DNA-positive cats and lower copy numbers of proviral DNA were seen in both vaccinated groups as compared to the placebo control group. The proportion of cats positive for proviral DNA was significantly different in Group A (RP-FeLV vaccine) than in Group C (Placebo) in 10 of 11 weeks. Statistical significance was found between Group B (canarypox-vectored FeLV vaccine) and Group C (Placebo) in 5 of the 11 weeks (Table 4).

All of the cats in the placebo control group had detectable levels of plasma RNA at some time during the post-challenge monitoring period (weeks 2 through 12 post-challenge). One cat in Group A (RP-FeLV vaccine) had a detectable level of plasma RNA at one timepoint, and three cats in Group B (canarypox-vectored FeLV vaccine) had detectable levels of plasma RNA at multiple timepoints during the post-challenge monitoring period. The proportion of cats positive for plasma RNA was significantly different in Group A (RP-FeLV vaccine) than in Group C (placebo) in 7 of 11 weeks. Statistical significance was found between Group B (canarypox-vectored FeLV vaccine) and Group C (placebo) in 5 of 11 weeks (Table 4).

**Figure 1:**
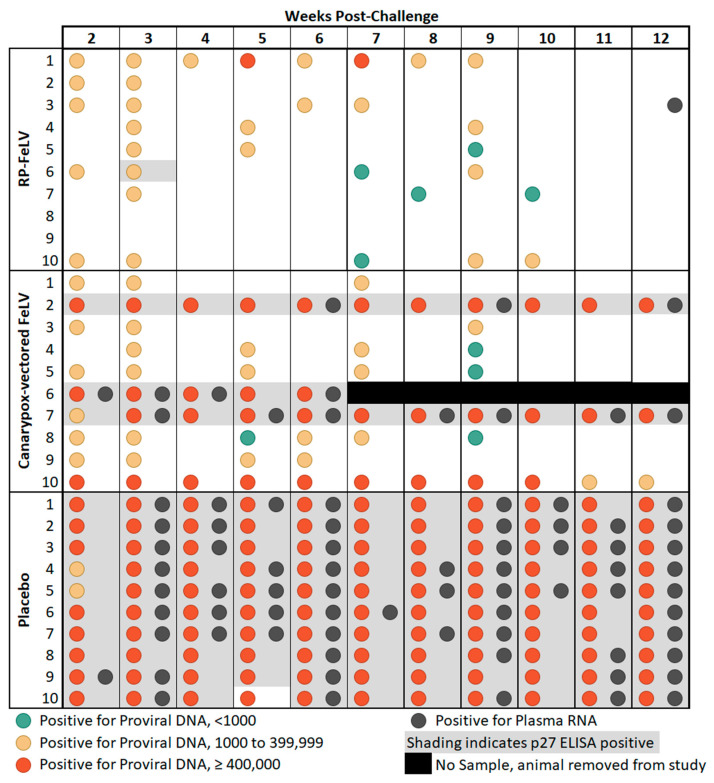
Comparison study summary of results. Blood samples were collected weekly from weeks 2 through 12 post-challenge and tested for FeLV p27 antigen by ELISA, proviral DNA by qPCR, and plasma RNA by RT-qPCR.

General agreement between proviral DNA copy number of at least 400,000 copies/mL and p27 antigenemia was observed as previously reported [8,30], as all 13 cats that were considered antigenemic had at least one sampling point with proviral DNA copy numbers of at least 400,000, and only two cats which had at least one sample with proviral DNA copy numbers of at least 400,000 were not considered antigenemic. There was one notable outlier, cat #10 in Group B, which had high levels of proviral DNA copy numbers on each weekly sampling from weeks 2 through 10 post-challenge yet tested negative for the FeLV p27 antigen on ELISA (Figure 1). Discordant FeLV test results have been previously reported in cats classified as regressive or thought to have a focal infection [8,9]. These findings may be related to the p27 antigen level not reaching the assay detection limits. In addition, the course of FeLV infection is influenced by the immune response and other host factors and can vary considerably between individual cats [6].

**Figure 2:**
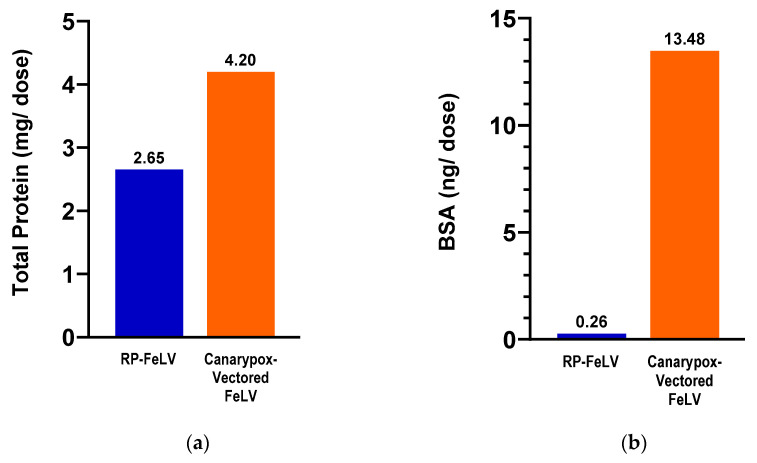
Commercial vaccines used in the comparison study. (**a**) Total protein as determined by BCA assay. (**b**) Bovine serum albumin as determined by BSA ELISA.

The vaccines used in the comparison study were tested for total protein and BSA content. The canarypox-vectored FeLV vaccine contained 1.5 times more total protein and 51 times more BSA compared to the RP-FeLV vaccine (Figure 2).

### 3.3. Field Safety Study

Among the 1657 doses administered, there were 45 adverse events deemed related to the vaccine (2.7% of doses administered), but none of the adverse events were considered serious. The most common reported adverse events were injection site pain (1.1% of doses) and lethargy (0.9% of doses). None of the cats were reported with injection site swelling.

## 4. Discussion

FeLV is a highly contagious retrovirus readily spread between cats in close physical contact and remains a significant feline pathogen despite the availability of vaccines. Being a retrovirus, a variety of the clinical signs of disease can be manifested, some years after infection; thus, it is very important that vaccines are able to greatly reduce viremia immediately following infection and prevent any insertion of proviral DNA sequences into the feline genome, especially as vaccination after infection is established as ineffective [31]. While several different FeLV vaccines are available, they differ in their design characteristics, especially regarding the use and type of adjuvants. Adjuvanted vaccines are usually formulated with inactivated whole FeLV virus or subunit proteins and are thought to induce an inflammatory response at the point of vaccination, which is necessary to stimulate an effective immune response when using an inactivated agent. While it has been suggested that inflammation may be linked to the development of feline injection site sarcomas (FISSs), no definitive causal relationship has been established [16]. In light of the concern regarding adjuvant use in cats, a non-adjuvanted, canarypox-vectored FeLV vaccine was developed to overcome the doubts regarding safety. However, while it has been used widely since its release, there remains debate as to whether this vaccine is as efficacious as adjuvanted vaccines [10,12,20,32,33,34]. Where efficacy studies have shown higher protection rates in cats vaccinated with adjuvanted vaccines versus nonadjuvanted vaccines, the adjuvanted vaccines are recommended in cats with a high risk for infection [16].

It is important to note that the composition of vaccines can differ significantly among various products, and even vaccines that share the same viral antigens or immunogenic components may have markedly different formulations due to variations in production techniques [35]. Besides adjuvants, another consideration when evaluating the safety of vaccines is the presence of other components, vaccine excipients, used in the manufacturing process. Vaccine excipients include the carrier fluids such as sterile water, saline, or solutions containing proteins; preservatives, antibiotics, thimerosal, and stabilizers; as well as adjuvants that enhance the vaccine’s efficacy. Furthermore, there may be residuals from the culture materials, such as growth media or bovine serum albumin. Some of these components can serve as potential allergens in select patients. BSA has been implicated in canine hypersensitivity reactions [36] and might also play a role in cats [16,37].

For these reasons, we aimed to develop a non-adjuvanted vaccine which is consistently as efficacious as the adjuvanted vaccines in terms of prevention of disease but based on a non-adjuvanted vaccine platform which delivers replicon RNA expressing the target antigen to cells. This replicon is based on the VEEV virus, though no VEEV is ever produced. The virus-like particle is used to deliver replicon RNA to the cells for the expression of the target protein. As the immunogenicity of the platform is dependent on which target protein is being expressed, to protect against FeLV, we chose to use the envelope glycoprotein present on the surface of FeLV virions and known to be a major protective immunogen [19,38,39]. In a series of studies, we demonstrated that the RP-FeLV vaccine was able to induce high levels of protection against antigenemia based on levels of p27 antigen, even when a single dose of RP-FeLV was compared to two doses of canarypox-vectored FeLV. Furthermore, in the comparison study, RP-FeLV-vaccinated cats not only had protection against persistent antigenemia, but they also exhibited reduced incidence and copy number/mL of proviral DNA and plasma RNA when compared to the placebo control cats. In contrast, 40% of the cats in the canarypox-vectored FeLV vaccine group demonstrated high levels of FeLV proviral DNA post-challenge, and 30% were repeatedly positive for FeLV plasma viral RNA. Furthermore, one of the canarypox-vectored FeLV vaccinates developed clinical disease attributed to FeLV infection. A statistically significant difference was demonstrated between each vaccine group and the control group for both the dose-ranging and comparison studies, yet not between the vaccine groups. An increased number of study animals would be required to detect a statistically significant difference between the RP-FeLV and canarypox-vectored FeLV vaccine groups. It is not uncommon for veterinary clinical studies to face the challenge of being underpowered (for the purpose of comparing two vaccine groups) due to small sample sizes, driven by the ethical imperative to minimize the number of animals used for welfare considerations. Given that *p*-values alone do not fully convey the nature of a clinical difference, the clinical effect should also be taken into consideration [40]. The difference between the RP-FeLV and canarypox-vectored FeLV vaccine groups could make a clinically meaningful difference for feline patients. The PCR results are clinically important because copy numbers of proviral DNA have been associated with how the disease course manifests in cats infected with FeLV. Using established cutoff values for proviral DNA, cats can be categorized as high positive or low positive and correlated with a shorter or longer survival time, respectively [8]. Forty percent of the cats vaccinated with the canarypox-vectored FeLV vaccine in the comparison study, including the cat that developed clinical disease, could be categorized as persistently high positive for proviral DNA (≥400,000 copies) and thereby at greater risk for a shorter survival time. In contrast, only one cat vaccinated with RP-FeLV exhibited proviral DNA copy numbers at ≥400,000 copies and only on two occasions in the first half of the post-challenge period. This cat had undetectable proviral DNA in each of the last three sampling periods.

As publications have questioned the use of immunosuppression of cats prior to challenge [21,34], in the two efficacy studies described herein, no immunosuppression was used, and high levels of antigenemia were detected in non-vaccinated control cats, demonstrating a robust and consistent challenge. In a previous comparative study between an adjuvanted vaccine and a canarypox-vectored FeLV vaccine [10], the relatively poor performance of the canarypox-vectored FeLV vaccine was suggested to be due to the immunosuppression of a cell-mediated response [34]. However, in the studies described herein in which no immunosuppression was used, the canarypox-vectored FeLV vaccine again demonstrated a lower level of protection against persistent antigenemia in comparison to 100% protection provided by the RNA particle FeLV vaccine. The vaccine potencies in the dose-ranging study (ranging from 3.6 × 10^7^ to 2.5 × 10^8^ RP/dose) represent the field dose (potency of commercial product).

Vaccine safety is an important consideration for pet owners and veterinarians when choosing a vaccine and adhering to vaccination protocols. While the overall incidence of adverse events associated with feline vaccinations has been demonstrated to be low [41], the administration of any biological product is not entirely risk free. Safety of the RNA particle FeLV vaccine was established in the field safety trial described here. Overall, the vaccine was well-tolerated in cats of all ages, including kittens as young as 8 weeks of age, which encompassed nearly half (45%) of the vaccinates. Of the 1657 doses administered, the most commonly reported adverse event was injection site pain (18 doses), followed by lethargy (15 doses).

Manufacturing vaccines on the RNA particle platform allows for production of vaccines with fewer excipients than other commercially available vaccine technologies. The RNA particle platform utilizes manufacturing processes that eliminate the need for antimicrobials and preservatives. Furthermore, harvesting the RNA particles does not require cell lysis, thereby minimizing cellular components and downstream processing steps to remove serum. Bovine serum albumin (BSA) is commonly used in veterinary and human vaccine production. The WHO has recommended that the BSA content for human vaccines to be less than 50 ng/dose [37]. There are no established guidelines for BSA levels in feline vaccines; however, it seems prudent to strive to reduce BSA levels as low as possible, as BSA has been associated with adverse events, including anaphylaxis, in dogs and cats [16,37].

The findings from the total protein and BSA evaluations indicate notable differences between the two FeLV vaccine formulations. In terms of their total protein content, there was a 37% lower concentration for the RNA particle FeLV vaccine versus the canarypox virus-vectored FeLV vaccine. An even more remarkable contrast was in the BSA content, with the RNA particle FeLV vaccine containing 98% less BSA as compared to the canarypox virus-vectored FeLV vaccine. Further post-marketing surveillance will be useful in determining the clinical significance.

## 5. Conclusions

Overall, these studies demonstrate that the RNA particle FeLV vaccine is safe and provides a high level of protection to cats in a nonadjuvanted formulation. The RNA particle FeLV vaccine, licensed under the name Nobivac^®^ NXT FeLV, is labeled for use in kittens as young as 8 weeks of age, has established a duration of immunity of at least 2 years [42], and will be a valuable asset in the ongoing control of FeLV.

## Figures and Tables

**Table 1:** Dose-ranging study design.

Treatment Group	Number of Cats	Vaccine	Vaccination Days
1	8	RP-FeLV 2.5 × 10^8^ RP/dose	0, 21
2	8	RP-FeLV 3.6 × 10^7^ RP/dose	0, 21
3	8	RP-FeLV 1.5 × 10^8^ RP/dose	21
4	8	Canarypox-vectored FeLV	0, 21
5	8	Placebo	0, 21

All cats were challenged on study days 49, 52, 54, and 56.

**Table 2:** Dose-ranging study results. Antigenemia is defined as three consecutive positive FeLV p27 results or five total positive results, consecutive or not, during the post-challenge monitoring phase. Preventable fraction is calculated as (percent of persistent antigenemia in controls—percent of persistent antigenemia in vaccinates)/percent of persistent antigenemia in controls × 100%. LL is lower limit; UL is upper limit.

Treatment Group	Vaccine	Cats Antigenemic	Preventable Fraction 95% Confidence Interval [LL, UL]
1	RP-FeLV 2.5 × 10^8^ RP/dose	0/8	100% [60%, 100%]
2	RP-FeLV 3.6 × 10^7^ RP/dose	0/8	100% [60%, 100%]
3	RP-FeLV 1.5 × 10^8^ RP/dose	1/8	86% [33%, 99%]
4	Canarypox-vectored FeLV	3/7	51% [−11%, 92%]
5	Placebo	7/8	-

**Table 3:** Comparison study results: antigenemia and preventable fraction. LL is lower limit; UL is upper limit.

Treatment Group	Vaccine	Cats Antigenemic	Preventable Fraction 95% Confidence Interval [LL, UL]
A	RP-FeLV	0/10	100% [69%, 100%]
B	Canarypox-vectored FeLV	3/10	70% [35%, 93%]
C	Placebo	10/10	-

**Table 4:** Comparison study results: proportion of cats positive for proviral DNA and RNA by PCR as compared to placebo-vaccinated cats. *p*-values were calculated using Fisher’s Exact Test (two-sided); statistical significance was declared at a *p*-value < 0.05 (indicated in bold type).

Weeks Post-Challenge	Proportion of Group Positive for Proviral DNA (*p*-Value)		Proportion of Group Positive for RNA (*p*-Value)	
	RP-FeLV vs. Placebo	Canarypox- Vectored FeLV vs. Placebo	RP-FeLV vs. Placebo	Canarypox- Vectored FeLV vs. Placebo
2	5 vs. 10 **(0.0325)**	9 vs. 10 (>0.9999)	0 vs. 1 (>0.9999)	1 vs. 1 (>0.9999)
3	8 vs. 10 (0.4737)	10 vs. 10 (>0.9999)	0 vs. 9 **(0.0001)**	2 vs. 9 **(0.0055)**
4	1 vs. 10 **(0.0001)**	4 vs. 10 **(0.0108)**	0 vs. 6 **(0.0108)**	1 vs. 6 (0.0573)
5	3 vs. 10 **(0.0031)**	8 vs. 10 (0.4737)	0 vs. 5 **(0.0325)**	1 vs. 5 (0.1409)
6	2 vs. 10 **(0.0007)**	6 vs. 10 (0.0867	0 vs. 10 **(0.0001)**	3 vs. 10 **(0.0031)**
7	4 vs. 10 **(0.0108)**	9 vs. 10 (0.2105)	0 vs. 1 (>0.9999)	0 vs. 1 (>0.9999)
8	2 vs. 10 **(0.0007)**	3 vs. 10 **(0.0031)**	0 vs. 3 (0.2105)	1 vs. 3 (0.5820)
9	5 vs. 10 **(0.0325)**	7 vs. 10 (0.2105)	0 vs. 9 **(0.0001)**	2 vs. 9 **(0.0055)**
10	2 vs. 10 **(0.0007)**	3 vs. 10 **(0.0031)**	0 vs. 4 (0.0867)	0 vs. 4 (0.0867)
11	0 vs. 10 **(<0.0001)**	3 vs. 10 **(0.0031)**	0 vs. 7 **(0.0031)**	1 vs. 7 **(0.0198)**
12	0 vs. 10 **(<0.0001)**	3 vs. 10 **(0.0031)**	1 vs. 10 **(0.0001)**	2 vs. 10 **(0.0007)**

## Data Availability

The datasets presented in this article are available upon request to the corresponding author.
